# Feasibility of a Tele-Assisted Home Exercise Program for Balance and Functional Mobility in Persons With Parkinson's Disease (TELEPORT-PD)

**DOI:** 10.1155/ijta/9936329

**Published:** 2025-06-05

**Authors:** Arnold Fredrick D'Souza, Dushyanth Babu Jasti, Rohini R. Rao, Manikandan Natarajan

**Affiliations:** ^1^Department of Physiotherapy, Manipal College of Health Professions, Manipal Academy of Higher Education, Manipal, Karnataka, India; ^2^Department of Neurology, Star Hospitals, Hyderabad, Telangana, India; ^3^Department of Data Science and Computer Applications, Manipal Institute of Technology, Manipal Academy of Higher Education, Manipal, Karnataka, India

**Keywords:** Parkinson's disease, postural balance, telehealth, telerehabilitation, videoconferencing

## Abstract

**Introduction:** Telerehabilitation can enhance delivery and acceptance of the home exercise program. This study evaluated the feasibility, safety, and signal of efficacy of a tele-assisted home exercise program for improving balance and functional mobility in persons with Parkinson's disease (TELEPORT-PD).

**Methods:** Eight participants underwent six sessions of physiotherapist-supervised and tailored multimodal exercise over smartphone videoconference through WhatsApp Messenger over 2 weeks. Feasibility was assessed through adherence and retention rates, and safety was assessed through self-reported adverse events. Balance was measured using the Berg Balance Scale (BBS) and Activity-specific Balance Confidence (ABC) scale, while functional mobility was assessed using Timed-Up-and-Go (TUG) test and 5-times Sit-To-Stand (5STS) test, at baseline and at the end of 2 weeks.

**Results:** The adherence and retention rates were 100%, and no serious adverse events were reported. Significant improvements were noted in the change scores of BBS (11.23 points; *p* = 0.014), ABC (14.21%; *p* = 0.014), TUG (-2.21 s; *p* = 0.007), and 5STS (-3.11 s; *p* = 0.007). All participants provided positive feedback and expressed their interest to continue the program.

**Conclusion:** TELEPORT-PD appears to be feasible, safe, and acceptable, while demonstrating improvements in balance and functional mobility.

**Trial Registration:** Clinical Trials Registry of India: CTRI/2022/09/045345

## 1. Introduction

Globally, Parkinson's disease (PD) is now the fastest growing neurological condition in terms of disability and mortality. The rapid increase in the prevalence of PD has affected countries across the socioeconomic spectrum. Currently India has the highest prevalence of PD among South Asian countries, followed by Pakistan and Bangladesh [1]. PD disproportionately affects older people [2]. The Longitudinal Aging Study of India has predicted that the elderly population in the country will increase threefold since the 2011 census [3], which may significantly strain the healthcare system in the future [4].

It is well documented that persons with Parkinson's disease (PwP) are at a higher risk of falling compared to age-matched healthy individuals. Alarmingly, this risk increases exponentially after the fourth decade of life [5]. Almost 70% of PwP are predicted to experience a fall annually [6, 7], and close to 90% of them will experience recurrent falls [8]. Falls have a devastating effect on the health-related quality of life of PwP [9]. Among the many fall risk factors identified, reduced postural balance, sluggish gait, and inadequate lower limb strength are some of the issues addressed by physiotherapy [7]. While PD rehabilitation is ideally a multidisciplinary effort, symptoms related to balance and gait dysfunction are only moderately managed by pharmacotherapy [10, 11]. Dopaminergic drugs have been found to worsen gait in the later stages of PD [12, 13]. Surgical management, such as deep brain stimulation, is effective but too expensive for most of the population [14]. On the other hand, exercise is relatively inexpensive, but its long-term effectiveness is limited primarily by poor adherence to regular exercise [15–17].

A physiotherapist prescribed, structured home exercise program (HEP) can promote physical activity and enhance the quality of life. Over the last two decades, HEPs have been extensively studied in PwP [18]. While the evidence on the effectiveness of unsupervised HEP for fall prevention among PwP is unclear when compared to center-based exercise [18, 19], a recent systematic review reported results comparable to those of center-based, physiotherapist-guided exercise training [20]. Additional benefits of HEPs include the potential for sustaining long-term, self-motivated exercise despite the limited availability of resources in most homes [20]. However, traditional HEPs need enhancement to unlock their long-term benefits for a broader range of PwP.

The immense challenges of the devastating COVID-19 pandemic have spurred numerous innovations in healthcare delivery and revitalized alternative healthcare models, with telehealth being one of the most significant [21]. The unprecedented circumstances during the pandemic led healthcare professionals and consumers to embrace telehealth as a convenient way to maintain access to care amid numerous public-health mandates that limited in-person interactions for nonemergencies [22]. Telerehabilitation (TR) has since gained renewed research and clinical interest, emerging as a recognized subspecialty within the physiotherapy profession [23]. The potential of low resource TR interventions to improve healthcare accessibility is particularly crucial in countries such as India [24, 25]. This is especially important given that the physiotherapy management of PD is a long-term process due to the chronic and progressive nature of the condition [26].

Home-based TR offers PwP the opportunity to continue their HEP under the remote guidance of a physiotherapist within the comfort and safety of their own homes. Although research in this area has been steadily increasing, only a handful of studies have been conducted in countries with lower socioeconomic development [27]. A prospective cohort study from India [28] and two studies from Brazil [29, 30] investigated the effects of smartphone video-call-assisted HEPs for PD management. Both studies reported that the intervention was feasible and safe, with potential improvements in motor abilities such as balance and functional mobility, as well as quality of life [28–30]. These benefits have been corroborated by multiple studies conducted in high-resource settings around the world [27]. These encouraging results highlight the need for population-specific research on a specially designed, cost-effective home-based TR program, which addresses the unique needs and challenges faced by PwP in low-resource settings.

This study had two objectives: The primary objective was to determine the feasibility and safety of the newly developed, low-resource tele-assisted HEP for balance and functional mobiliTy in PwP (TELEPORT-PD), whereas the secondary objectives were to explore the efficacy signals for balance and functional mobility. Additionally, the study is also aimed at understanding the participants' perspectives toward the program.

## 2. Methods

### 2.1. Study Design

This feasibility study was conducted between October 2023 and March 2024 after receiving approval from the Institutional Ethics Committee (IEC1: 109/2022). All the participants agreed to participate in the study and provided written informed consent before commencement.

### 2.2. Participants

Given the exploratory nature of the study, sample size calculations were not performed. Eight participants were recruited from the neurology department of a tertiary care hospital in southern India.

The participants were adults diagnosed with PD by a neurologist on the basis of the United Kingdom Parkinson's Disease Society Brain Bank criteria. Participants were within Hoehn and Yahr Stages 1–3, on stable dopaminergic medications for at least 2 weeks and independently ambulant without the need for any assistive device. They were also required to have access to the internet via a smartphone, tablet, or computer. A functionally independent informal caregiver had to be present at home during all sessions to ensure the safety of the participant and provide any necessary assistance during the session. The study excluded individuals who presented with significant cognitive impairment (Montreal Cognitive Assessment (MoCA) score less than 21) [31], high fall risk (Pull test score greater than two), significant visual, speech, or hearing impairments, coexisting medical conditions or recent surgery that might impede participation in a structured exercise program, unstable fractures of the spine or limb bones, simultaneous participation in any other exercise or physical activity program(s), and those with motor fluctuations or dyskinesia.

### 2.3. Procedure

**Figure 1:**
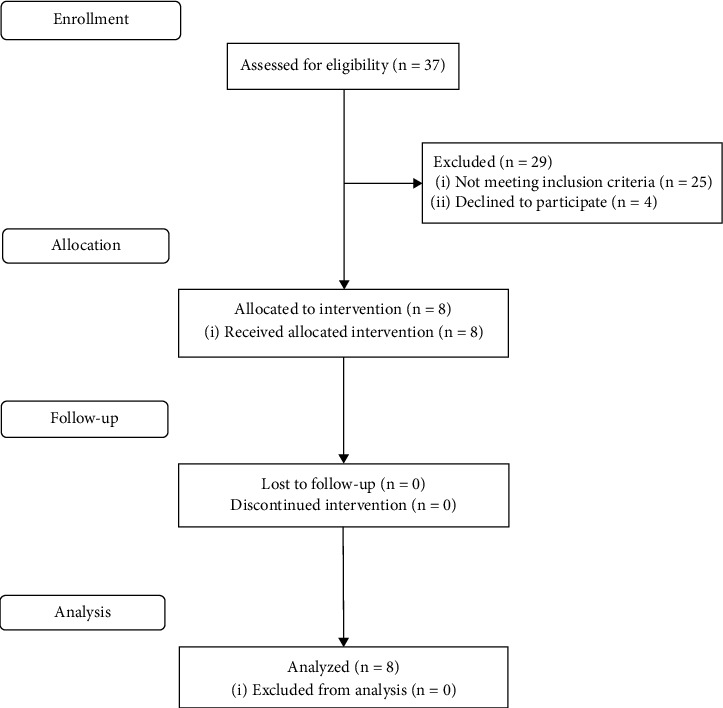
Participant flow chart.

The participant flowchart has been depicted in Figure 1. Thirty-seven PwP were screened, eight of whom were enrolled in the study based on the eligibility criteria. Demographic characteristics were documented, and clinical data, including time since diagnosis, Hoehn and Yahr scale stage, cognitive level using the MoCA, Pull test score, and Levodopa-equivalent daily dose (LEDD) based on reported medication, were recorded. A baseline assessment was conducted to evaluate balance and functional mobility before the initiation of the treatment program. Balance was measured using the Berg Balance Scale (BBS) [32] and the Activity-specific Balance Confidence (ABC) scale [33], while functional mobility was assessed using the Timed-Up-and-Go (TUG) [34] and 5-times Sit-To-Stand (5STS) [35] tests. The MoCA, BBS, and ABC scales were used with permission for the purposes of this study.

The BBS is a 14-item list with each item consisting of a five-point ordinal scale ranging from 0 to 4, with 0 indicating the lowest level of function and 4 the highest level of function [32]. The ABC scale is a 16-item questionnaire to assess the degree of confidence of the person in their balance associated with daily life. The score is a percentage where higher values represent a better outcome [33]. The TUG measures the time that a person needs to stand up from a chair, walk a 3-m distance, turn around, and sit back on the chair [34]. For the 5STS, participants are instructed to sit on a chair with their back resting against the back of the chair while crossing their arms over their chest. The participants had to stand up and sit down five times as soon as possible [35]. The psychometric properties of BBS [36], ABC [37], TUG [37], and 5STS [38] have been established in populations with PD.

A physiotherapist visited each participant's home to conduct an in-person familiarization session, training the participants and their caregivers in setting up their devices for WhatsApp video calls and designating a suitable area at home for conducting the sessions. Troubleshooting advice was provided upon request. The exercise program was demonstrated in person to ensure that the participants understood all the exercises, received detailed instructions, and had any questions answered. Additionally, each participant received an exercise instruction manual and videos and was asked not to engage in any other exercise-based interventions during the study period.

Participants were engaged in six sessions on nonconsecutive days over a 2-week period. All sessions were conducted during the “on” phase, and the medication dosage remained constant throughout the study period.

**Figure 2:**
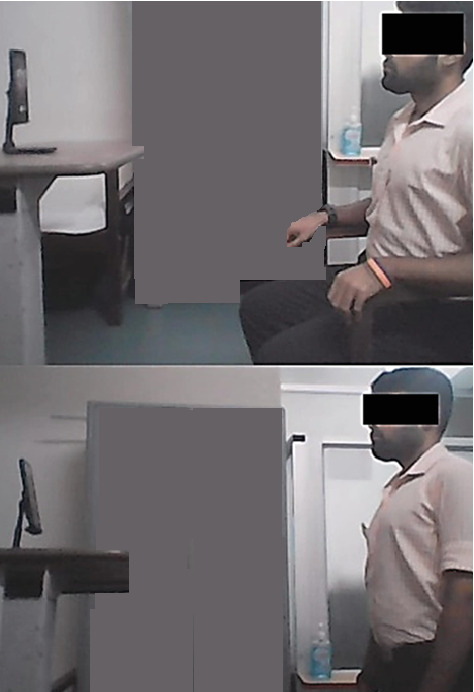
Smartphone placement in sitting and standing position.

All sessions were conducted through real-time remote guidance from the physiotherapist in a designated private room at the hospital over WhatsApp video calls. The physiotherapist used a 5G-enabled Android smartphone (Galaxy S20 FE 5G, Samsung Electronics, South Korea) with an active 5G data plan, with the participant on the other end in their home. The smartphone was positioned in portrait orientation on a generic mobile phone stand placed on a height adjustable table at the physiotherapist's eye level, whether in seated or standing position (Figure 2). During the exercise demonstrations, the physiotherapist was positioned at least 5–6 ft from the smartphone which ensured that both the outstretched arms and lower limbs were fully visible to the participant.

The exercise sessions began in a seated position and then progressed to standing. A sturdy chair was mandatory for all participants to ensure safety during seated and supported-standing exercises. The physiotherapist demonstrated each exercise and performed them alongside the participant, frequently soliciting feedback. If discomfort or pain was reported, the exercise was immediately stopped and either modified or omitted entirely before proceeding to the next exercise.

Three sessions were conducted every week on nonconsecutive days over 2 weeks. Each session lasted between 30 and 45 min. The physiotherapist ensured that the caregiver was present throughout each session to maintain the participant's safety and provide any necessary assistance. If the caregiver was unavailable, the session was rescheduled to a different time.

## 3. TELEPORT-PD

The TELEPORT-PD protocol was developed through an international e-Delphi consensus, involving an interprofessional panel of experts in PD management. The program was specifically designed for use in low-resource settings.

It is a multimodal intervention incorporating balance, functional mobility, endurance, strength, flexibility training, and relaxation techniques, tailored to each participant. Exercises were performed in seated and standing positions, using available equipment at the participant's home. Water bottles were used for upper limb strength training. All exercises were conducted at moderate intensity or higher, as described by the Borg's Rating of Perceived Exertion (RPE) scale. If a participant reported difficulty, the intensity was reduced; if the exercises were reported to be easy, progression was made in terms of repetition and duration. Endurance, strength, balance, and functional mobility training were performed at least 2–3 days a week on nonconsecutive days, and each domain was performed for at least 10 min. Flexibility and relaxation techniques could be performed daily with each domain lasting at least 5 min. The total session duration was at least 30 min. A detailed list of exercises within each domain, their dosage, and additional information is provided in Table 1.

### 3.1. Outcome Assessment

The feasibility of the program was determined by the adherence rate (number of completed sessions), retention rate (number of dropouts), challenges encountered, and alternative solutions adopted. Safety was evaluated by the number of participant-reported adverse events. Balance and functional mobility were evaluated at baseline and at the end of 2 weeks. The outcome measures were administered at the participant's home by another senior physiotherapist who was not involved in the study. All evaluations were carried out during the “on” phase.

After the program had concluded, semistructured interviews were conducted with all participants to gather their experiences and perspectives on the program. An expert-validated in-depth interview guide was used to collect feedback, including specific comments on the program's content and its delivery. The interviews were recorded with participants' consent, and verbatim transcripts were prepared. Non-English responses were translated into English by a bilingual native speaker. All potential identifiers were removed before data transcription.

### 3.2. Statistical Analysis

Data analysis was performed using EZR statistical software (Version 1.68). Demographic characteristics were summarized using basic descriptive statistics and are presented as means with standard deviations or medians with interquartile ranges (IQRs). The feasibility and safety outcomes are reported as frequencies and percentages. The Shapiro–Wilk test was conducted to assess data normality. To evaluate changes between pre and posttest scores for the BBS, ABC scale, TUG, and 5STS tests, the Wilcoxon signed-rank test was employed. Statistical significance was determined at *p* < 0.05. Qualitative data from interviews were analyzed through inductive thematic analysis, with descriptive codes identified via repeated analysis.

## 4. Results

### 4.1. Participant Characteristics

The demographic and clinical characteristics of the study participants are presented in Table 2. The participants were predominantly male (*n* = 6, 75%) and most participants reported at least an undergraduate level of education (*n* = 6, 75%). The participants were between Stages 2 and 3 on the Hoehn and Yahr scale with Pull test scores not greater than two. The mean duration of PD was 5 years, and the median LEDD was 394.5 mg.

### 4.2. Feasibility Metrics

All participants completed the six TELEPORT-PD sessions, without any dropouts. Seven out of 48 sessions were disrupted by poor mobile network reception at the participant's end. Telephone calls were made to the caregiver during such situations to decide the next course of action. Whenever 75% or more of the program content for the session was completed prior to the disruption, the remaining part of the session was completed via telephonic supervision with the help of a caregiver. The participants were encouraged to continue the exercises with instructions provided by the physiotherapist over the phone and the caregiver providing feedback, thus allowing the participant to complete the remainder of the program.

In situations where disruptions occurred during the early part of the program, the session was rescheduled to the next day, and the entire session started from the beginning and completed. Additionally, two sessions were rescheduled to the next day, as the caregiver was unavailable during the initial scheduled day.

All the participants (*n* = 8) chose smartphones as their preferred device, with the WhatsApp Messenger mobile application (Meta Platforms Inc., California, United States) being the most favored tool for videoconferencing sessions. Two participants used a 5G-enabled Apple and Android smartphone, respectively, five participants used a 4G-enabled Android smartphone, and one participant used a 3G-enabled Android smartphone. All smartphones had a display size of 5.5 in. or larger and at least a 720 p front-facing camera. Most participants (*n* = 6) connected via individual mobile internet networks, whereas the remaining two used a home Wi-Fi connection.

The average session duration was 34 min. Except for one participant, all the participants exercised in the morning. All exercises were performed as described. Partial squats were the least comfortable exercise, followed by trunk mobility exercises, particularly forward bending. Modifications were made to prevent or minimize pain and discomfort. No special modifications were requested by participants who did not report musculoskeletal pain.

### 4.3. Safety Metrics

While no participants reported any serious adverse events during the study period, three participants reported low back pain, and one reported mild knee pain after the session. Pain levels were less than seven on the numerical pain rating scale and were completely resolved by the next session. Since the sessions were conducted on alternate days, there was a 48-h interval before the next session, during which participants were advised to minimize strain on the affected region. They were also advised to use a hot water bag over the area for pain relief every 4–6 h for approximately 15–20 min. Exercises were modified for these participants by reducing repetitions or sets, increasing rest periods, incorporating more frequent breaks, minimizing lower back strain by avoiding or reducing the range of motion during forward bending, and avoiding breath-holding during exercise training. There were no reports of pain after these modifications were introduced. The details of feasibility and safety metrics for the TELEPORT-PD are given in Table 3.

### 4.4. Signal of Efficacy

The Wilcoxon signed-rank test was used to ascertain the signal of efficacy for all outcome measures. At the end of 2 weeks, statistically significant improvements were noted in all outcome measures: BBS (mean difference of 11.23 points, *p* = 0.014), ABC (mean difference of 14.21%, *p* = 0.014), TUG (mean difference of -2.21 points, *p* = 0.007), and 5STS (mean difference of -3.11 s, *p* = 0.007). The baseline and posttreatment values of all the outcomes are given as median and IQR in Table 4.

### 4.5. Qualitative Findings

Participants described the program as “simple” and “motivating” and reported to be “happy” at the end of the program. In summary, all the participants reported positive experiences with TELEPORT-PD and were satisfied with the program's content and conduct. The most common challenges faced were due to the relative novelty of exercising via remote guidance through video calls on WhatsApp Messenger. Participants emphasized the importance of the in-person familiarization session conducted in their home before starting the tele-sessions to allow for a smoother transition. They also highlighted several advantages of the program, including its ease, customizability, convenience, and flexibility. Some participants recommended modifications, such as increasing the treatment duration and the number of sessions. The participants also expressed interest in continuing their participation in the program.

## 5. Discussion

This study assessed the feasibility and safety of TELEPORT-PD, a multimodal, home-based exercise program designed for community-dwelling early-to-midstage PwP. TELEPORT-PD was developed through an international, interprofessional e-Delphi consensus and delivered via WhatsApp video calls, with physiotherapist-led guidance and supervision throughout each session. To ensure participant safety during the exercise training, the program required the presence of a caregiver to assist and monitor the participant.

An adherence and retention rate of 100% was achieved for all participants, which could be the result of a combination of factors. First, the treatment schedule was flexible, and the participants were able to reschedule sessions that were missed or interrupted for technical or personal reasons. The active participation of the caregiver allowed for easier communication between the physiotherapist and the participant, in addition to fostering a sense of familiarity and ensuring safety. The technical challenges were primarily due to internet connectivity issues, and these were comparable to reports in other TR studies [28, 39]. High-speed internet is a prerequisite for video-based communication [40], especially since this was the selected mode of exercise instruction. However, because mobile internet is the most common method of accessing the internet in India, the inherent limitations of wireless network reception in suburban and rural areas lead to buffering, lag, and disconnections [41]. This was remedied by ensuring that the participants were seated, and a phone call was made to the caregiver on an alternate number. Similar methods have also been employed in other studies [28, 39].

Except for musculoskeletal pain, none of the participants reported serious adverse events. The episodes of musculoskeletal pain resolved without the need for any medical intervention. Exercising on alternate days must have provided enough rest period to allow for the symptoms to spontaneously resolve. In addition, the hot water bag application prescribed by the physiotherapist could have reduced the soreness resulting from exercise from the session. The participants who experienced musculoskeletal pain had a history of low back pain or knee pain prior to participation. Exercise intensity was lowered to accommodate this, but on those who presented with recurrence, exercise was discontinued. These findings suggest that participant selection and exercise customization are essential components that allow for safe and effective administration of exercise programs [42, 43].

This study additionally examined the signal of efficacy for balance and functional mobility, as well as the perspectives of PwP toward the program. Two weeks of training via the TELEPORT-PD intervention resulted in statistically significant improvements in balance and functional mobility among PwP. The findings of this study are congruent with similar studies conducted around the world that used different home-based TR approaches for fall prevention in PwP [27, 30, 39]. More importantly, despite the use of a low-resource TR delivery alternative to provide a similar exercise-based intervention, TELEPORT-PD was still capable of enhancing balance and functional mobility in a relatively short period of time. This finding is encouraging and highlights the need for more research focused on enhancing the accessibility of preexisting effective treatments via novel delivery methods.

The marked improvement in balance and functional mobility could be attributed to the active supervision and guidance provided by the physiotherapist [19, 44]. The personalization of the program might have increased participant engagement and encouraged them to continue performing the exercise even after the program had concluded. The active involvement of the participant's caregiver throughout each session must have played a significant role in improving the program's effectiveness by increasing the participant's confidence through the maintenance of safety and ensuring a sense of familiarity [45, 46]. The lack of need to procure special equipment or any advanced technology could have increased the acceptability of TELEPORT-PD among PwP. WhatsApp Messenger is the most ubiquitous personal messaging software in India, and all participants were familiar with the basic functionality of the application because of its daily frequent use before study participation. This familiarity might have assisted participants in acclimating to TR.

All participants reported positive experiences with the TELEPORT-PD intervention. The real-time guidance provided by the physiotherapist and the involvement of caregivers, combined with the comfort of exercising at home, likely contributed to their favorable perceptions. Another key factor that may have enhanced acceptability was the program's individualized approach, which tailored exercises to each participant's specific needs and abilities. Personalized exercise programs have been shown to improve compliance by aligning with participants' treatment goals. Some participants, however, found the exercise duration to be too short. This may have been due to the physiotherapist's cautious approach, given the nature of the study, with safety being prioritized. Despite this, participants expressed a strong desire to continue the program if given the opportunity. These findings align with previous studies where initial apprehension toward novel interventions diminished as participants became familiar with the approach [27, 47, 48].

### 5.1. Limitations

The small sample size owing to the nature of the study design will limit the generalizability of the findings. Another key limitation arising from the lack of funding was the lack of advanced tools for objectively quantifying functional improvements. Additionally, variability in devices and network technology used to conduct the sessions may have influenced the effectiveness of the intervention. The exercise duration was not standardized, as the study was exploratory in nature, and this variation could have impacted the outcomes. Furthermore, the long-term effects of the TELEPORT-PD program remain unclear due to the absence of follow-up assessments.

### 5.2. Implications

This study adds to the pool of clinical research data to support the use of a low-resource, real-time, physiotherapist-supervised and guided home-based TR intervention for balance and functional mobility retraining for PwP. The findings from this feasibility study have been used to develop an ongoing 6-week randomized controlled trial with a 3-month follow-up.

## 6. Conclusion

TELEPORT-PD, a recently developed, low-resource, physiotherapist-supervised and guided, home-based TR intervention, appears to be feasible and safe for early-to-midstage PwP. Additionally, it seems to improve balance and functional mobility in the short term. Finally, PwP expressed positive opinions about the program and recommended its use.

## Figures and Tables

**Table 1:** Details of the TELEPORT-PD protocol.

**Domain**	**Exercises**	**Frequency**	**Intensity**	**Time**	**Progression criteria**
Aerobic training	Brisk walking, jogging, static cycling, and walking on a treadmill	At least 2–3 nonconsecutive days per week	At least moderate (according to Borg RPE scale)	At least 10 min	Increase duration and speed
Resistance training	Front raise (shoulder flexion), side raise (shoulder abduction), elbow flexion, overhead triceps extension, overhead shoulder press, wall or counter push-ups, modified plank (core strengthening), hip abduction, hip flexion, hip extension, knee extension, and knee flexion	At least 2–3 nonconsecutive days per week	At least moderate (according to Borg RPE scale)	At least 10 min	Increase resistance and repetitions
Balance training	Weight shifts in sitting, standing with feet together, weight shifts in standing, tandem standing, single leg standing, standing on a cushion, reaching in different directions, stepping in different directions, walking along a line, tandem walking, figure of eight walking, obstacle walking, and dual-task gait training	At least 2–3 nonconsecutive days per week	At least moderate (according to Borg RPE scale)	At least 10 min	Increase duration, reduce hand support, keep eyes closed, stand on compliant surface, and double to single limb support
Functional mobility training	Bridging, quadruped, kicking in different directions, sit to stand, partial squat, marching in place, walking with large steps, walking with greater arm swing, and audiovisual cue-assisted gait training	At least 2–3 nonconsecutive days per week	At least moderate (according to Borg RPE scale)	At least 10 min	Increase duration and speed
Flexibility training	Trunk flexion, trunk rotation, shoulder stretch, neck muscles stretch, upper and lower limb stretch, and active range of motion for neck, trunk, and limbs	Daily	Not applicable	At least 5 min	Increase hold duration and repetitions
Relaxation techniques	Deep breathing and progressive muscle relaxation	Daily	Not applicable	At least 5 min	Not applicable

*Note:* Termination criteria: fatigue, pain, or discomfort; safety considerations: Use support as required. Endurance and strength training were performed on the same day. Balance and functional mobility training were performed on the same day. A tailored exercise plan was created for each participant by selecting exercises that accommodate their specific needs and ability level.

Abbreviation: Borg RPE = Borg Rating of Perceived Exertion.

**Table 2:** Demographic and clinical characteristics of study participants (*n* = 8).

**Parameters**	**Frequency ** **n** ** (%)**	**Mean (SD)**
Age in years	—	67.5 (6.4)
Gender		
• Male	6 (75)	
• Female	2 (25)	
Marital status		
• Married	8 (100)	
• Single	0 (0)	
Educational status		
• Primary school	1 (12.5)	
• High school	1 (12.5)	
• Undergraduate	4 (50.0)	
• Postgraduate or higher	2 (25.0)	
Area of residence		
• Suburban	4 (50)	
• Rural	4 (50)	
Years since diagnosis of Parkinson's disease	—	5.31 (4.9)
Levodopa-equivalent daily dose (LEDD)		
• Low (< 600 mg)	5 (62.5)	
• High (≥ 600 mg)	3 (37.5)	
Hoehn and Yahr scale		
• Stage 2	2 (25.0)	
• Stage 2.5	3 (37.5)	
• Stage 3	3 (37.5)	
Montreal Cognitive Assessment score	—	24.4 (3.3)

**Table 3:** Feasibility and safety metrics for the TELEPORT-PD (*n* = 8).

**Feasibility metrics**	**Value**
Adherence rate (%)	100
Retention rate (%)	100
Session duration (minutes); mean (SD)	34 (8)
Challenges faced (*n*)	
• Technical	07
• Personal	02
Safety metrics	
Serious adverse events (*n*)	0
Adverse events (*n*)	04
• Low back pain	03
• Knee pain	01

**Table 4:** Signal of efficacy for balance and functional mobility (*n* = 8).

**Outcome measures**	**Baseline median (Q1, Q3)**	**Posttest median (Q1, Q3)**	**p**
Berg Balance Scale scores	40.5 (38.8, 44.0)	53.0 (52.8, 53.2)	0.014
Activity-specific Balance Confidence Scale (%)	62.8 (54.8, 68.3)	76.9 (70.2, 85.2)	0.014
Timed-Up-and-Go (in seconds)	13.8 (12.8, 06.1)	12.3 (09.4, 13.1)	0.007
5-times Sit-To-Stand (in seconds)	15.0 (13.3, 18.6)	13.1 (12.2, 13.4)	0.007

## Data Availability

The data that support the findings of this study are available from the corresponding author upon reasonable request.
